# Sarcoidosis Mimicking Metastatic Disease: Multisystem Involvement With Osseous Manifestations

**DOI:** 10.7759/cureus.98390

**Published:** 2025-12-03

**Authors:** Filipa Canedo, Inês Duarte, João Rodrigues, Maria Inês Carvalhinho, Alexandra Borba

**Affiliations:** 1 Pulmonology, Hospital Santa Marta, Unidade Local de Saúde de São José, Lisbon, PRT; 2 Anatomical Pathology, Unidade Local de Saúde de São José, Lisbon, PRT

**Keywords:** bone involvement, immunosuppressive therapy, malignancy suspicion, multisystem disease, sarcoidosis

## Abstract

Sarcoidosis is a chronic granulomatous disease of unknown etiology that can affect multiple organs, most frequently the lungs, lymph nodes, skin, and eyes. Osseous involvement is a rare manifestation, typically affecting small bones, although axial skeleton lesions may also occur. We report the case of a 42-year-old woman with no relevant past medical history who presented with fatigue, night sweats, diffuse joint pain, and low back pain. Fluorine-18 fluorodeoxyglucose positron emission tomography/computed tomography demonstrated multiple hypermetabolic pulmonary nodules, predominantly in the right upper lobe, associated with bilateral hilar, mediastinal, and supraclavicular lymphadenopathy, as well as focal uptake in the right iliac bone, sacrum, and L4 vertebral body. Given the imaging pattern and metabolic activity, disseminated malignancy was initially suspected. Histopathological examination of mediastinal lymph node and iliac bone biopsies revealed noncaseating granulomatous inflammation. Laboratory results showed elevated serum angiotensin-converting enzyme, negative interferon-gamma release assay, and negative autoimmune serologies. Following a multidisciplinary discussion, a diagnosis of sarcoidosis was established. The patient was treated with systemic corticosteroids followed by methotrexate, with significant clinical improvement. This case highlights an uncommon presentation of multisystemic sarcoidosis with axial skeletal involvement and imaging findings mimicking metastatic disease, underscoring the importance of histological confirmation and multidisciplinary assessment in establishing an accurate diagnosis and guiding management.

## Introduction

Sarcoidosis is a chronic multisystem granulomatous disorder of unknown etiology, most frequently involving the lungs and intrathoracic lymph nodes, although virtually any organ may be affected [1]. Although the precise trigger remains unclear, several environmental and occupational exposures, including bioaerosols, mold, and inorganic dusts, have been associated with increased disease risk. Histologically, sarcoidosis is characterized by noncaseating epithelioid granulomas in the absence of identifiable causes. The disease course is heterogeneous, ranging from asymptomatic incidental findings to progressive multiorgan involvement with significant morbidity. A well-defined acute presentation, known as Löfgren syndrome, is characterized by erythema nodosum, bilateral hilar lymphadenopathy, and acute arthritis and generally has a favorable prognosis [2].

Osseous involvement is uncommon, estimated to occur in approximately 3-13% of patients depending on the cohort and diagnostic method [3,4]. Classically, it affects the small bones of the hands and feet, but axial skeleton lesions, particularly involving the pelvis, sacrum, and vertebrae, are increasingly recognized through advanced imaging techniques [5,6]. Fluorine-18 fluorodeoxyglucose positron emission tomography/computed tomography (¹⁸F-FDG PET/CT) and MRI have substantially improved detection. FDG-PET/CT highlights areas of increased glucose metabolism, which may be seen in both inflammatory and malignant processes, often revealing metabolically active or lytic lesions not visible on conventional radiography or low-dose CT [6-8]. Because such lesions may closely resemble metastatic disease both morphologically and metabolically, histological confirmation is needed. The integration of clinical, radiological, and pathological findings within a multidisciplinary discussion (MDD) remains the reference standard for establishing the diagnosis [7-9].

Treatment is required only in a subset of patients, with the goal of preventing long-term complications and improving quality of life [10]. Management must be individualized, ranging from surveillance in asymptomatic cases to systemic corticosteroids and immunosuppressive agents in patients with significant or progressive disease [10,11].

We present a case of multisystem sarcoidosis with pulmonary, nodal, and axial skeletal involvement that closely mimicked disseminated malignancy, emphasizing the importance of histological confirmation and multidisciplinary assessment in complex presentations.

## Case presentation

A 42-year-old woman, a nonsmoker with a personal history of allergic rhinitis, was referred for evaluation of persistent fatigue impacting daily activities, along with night sweats, diffuse joint pain, and low back pain, with approximately six months of symptom evolution. She denied fever, weight loss, respiratory symptoms, or recent infections. The chronic time course and clinical presentation were not suggestive of Löfgren syndrome. Family history was notable for psoriasis in multiple first-degree relatives and sarcoidosis in a cousin. Physical examination revealed tenderness over the right sacroiliac region, with no other significant abnormalities.

**Figure 1:**
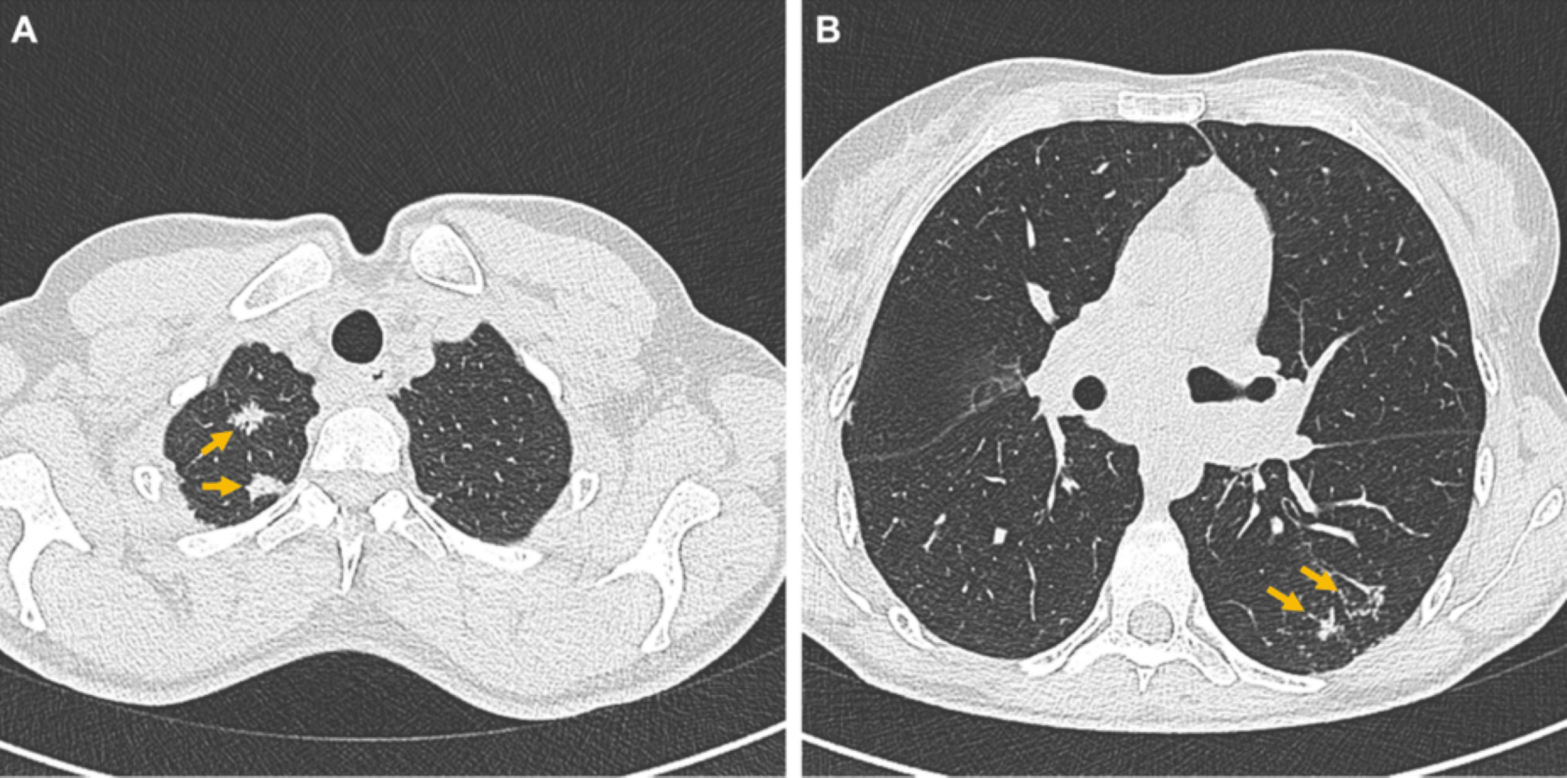
Axial chest CT images showing (A) two spiculated nodules in the right upper lobe and (B) irregular micronodules in the left lower lobe.

Chest CT demonstrated multiple spiculated pulmonary nodules, the largest located in the right upper lobe, measuring 15 mm and 12 mm, along with irregular micronodules in the left lower lobe (Figure 1A, 1B). A conglomerate of mediastinal lymph nodes was also identified.

**Figure 2:**
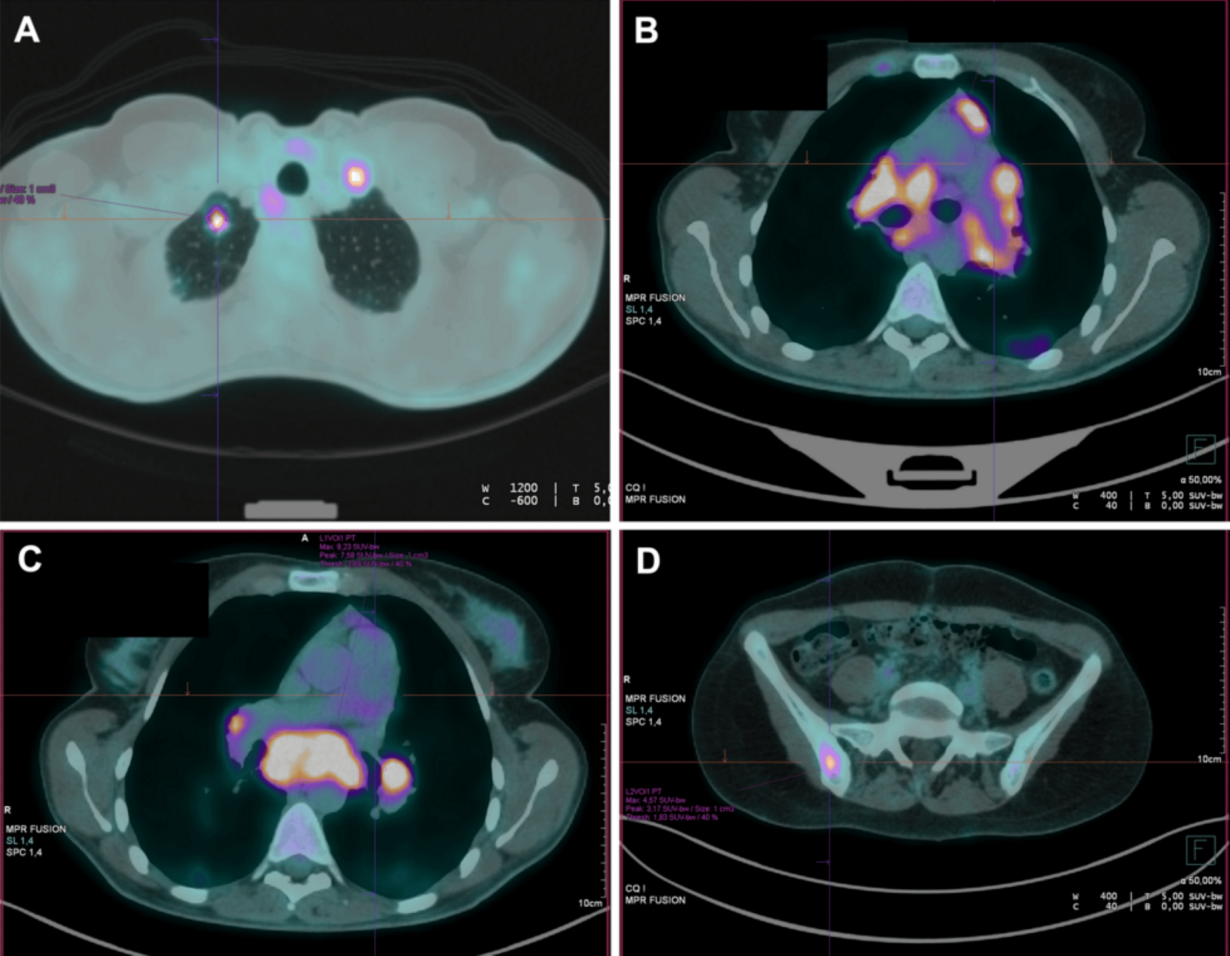
Baseline ¹⁸F-FDG PET/CT images showing (A) hypermetabolic pulmonary nodules in the right upper lobe (SUVmax = 9.78), (B, C) mediastinal and hilar lymphadenopathy (SUVmax = 9.23), and (D) intense focal uptake in the right iliac bone (SUVmax = 11.22). ¹⁸F-FDG PET/CT: fluorine-18 fluorodeoxyglucose positron emission tomography/computed tomography

Subsequent ¹⁸F-FDG PET/CT revealed multiple hypermetabolic pulmonary nodules, predominantly in the right upper lobe (SUVmax = 9.78), associated with bilateral hilar, mediastinal, and left supraclavicular lymphadenopathy. Increased uptake was also observed in the right iliac bone (SUVmax = 11.22), sacrum, and L4 vertebral body (SUVmax = 8.56) (Figure 2A-2D). This pattern raised concern for metastatic malignancy, although lymphoma and granulomatous infection were also considered in the initial differential diagnosis.

**Figure 3:**
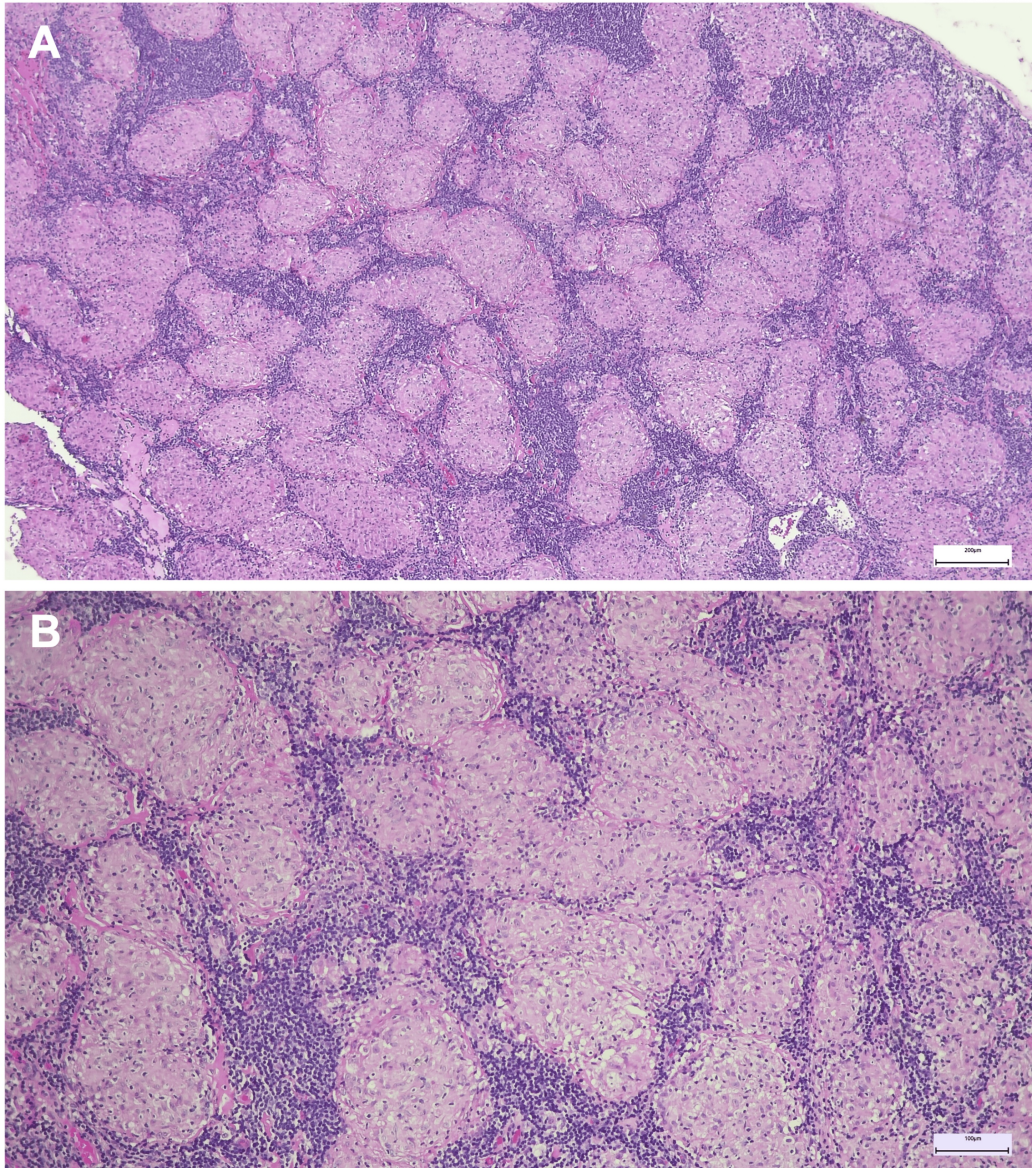
Lymph node biopsy showing replacement of normal architecture by multiple epithelioid granulomas, some coalescent, without necrosis. Findings consistent with non-necrotizing granulomatous lymphadenitis (A: H&E, ×50; B: H&E, ×100).

**Figure 4:**
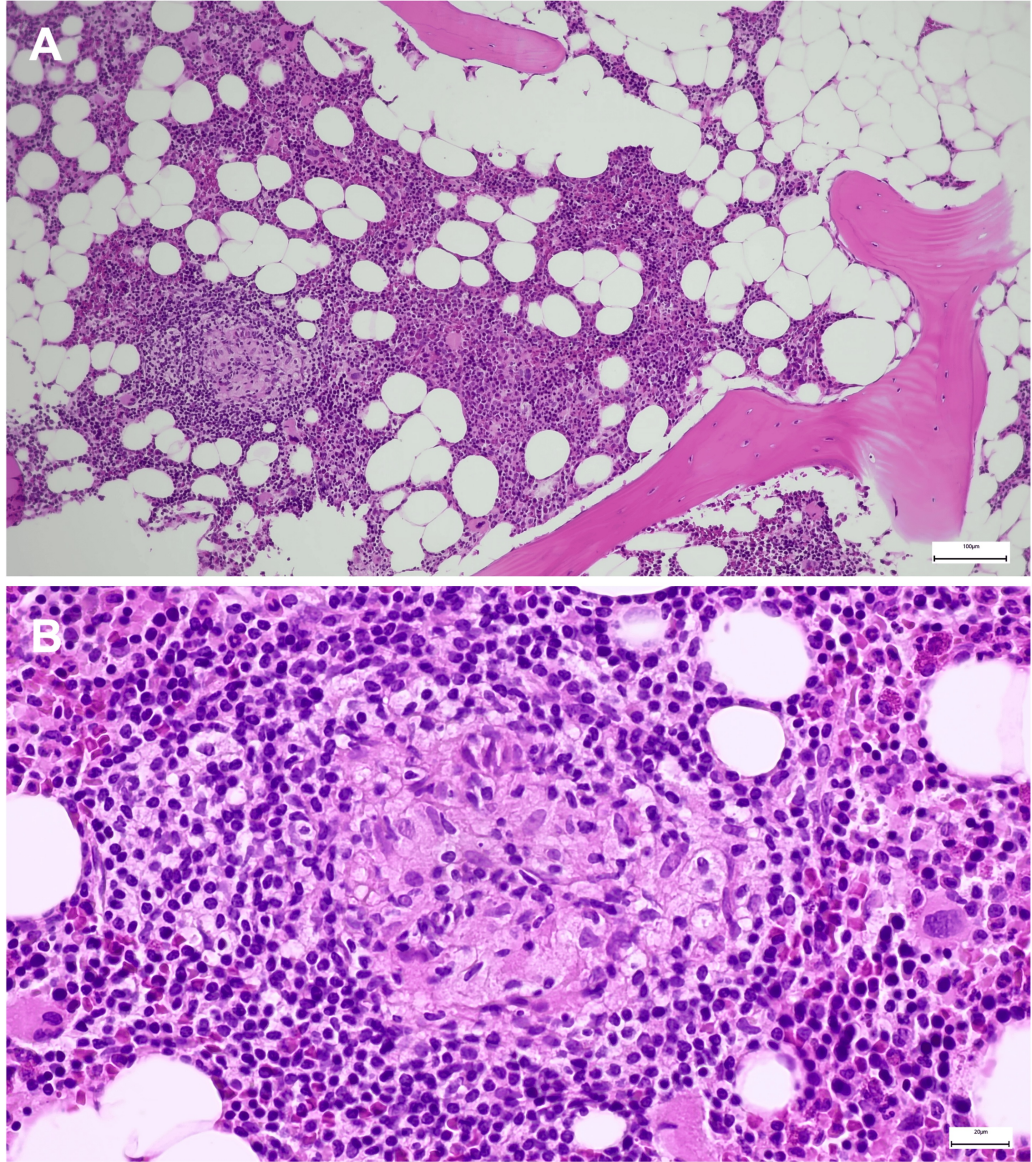
(A) Bone marrow biopsy showing infiltration by non-necrotizing granulomatous inflammation (H&E, ×100). (B) Detail of an epithelioid granuloma without necrosis (H&E, ×400).

Given the extent of nodal and osseous involvement on PET/CT, the case was discussed at a multidisciplinary team meeting. Both conventional endobronchial ultrasound-guided biopsy (EBUS) and cryo-EBUS were considered as minimally invasive diagnostic options. However, mediastinoscopy was preferred to obtain a larger tissue sample with preserved nodal architecture, considered necessary for a definitive histological diagnosis. Mediastinal lymph node biopsy revealed complete architectural effacement by noncaseating epithelioid granulomas without necrosis or multinucleated giant cells, consistent with granulomatous lymphadenitis (Figure 3A, 3B). Due to the marked FDG uptake in bone lesions, a CT-guided biopsy of the right iliac lesion was subsequently performed, demonstrating non-necrotizing granulomatous infiltration of the bone marrow (Figure 4A, 4B).

Laboratory evaluation showed elevated serum angiotensin-converting enzyme (ACE) at 70 U/L (reference range: 8-52 U/L). The interferon-gamma release assay was negative, the autoimmune panel was unremarkable, and routine laboratory tests, including complete blood count, inflammatory parameters, serum calcium, and liver and renal function, were within normal limits (Table 1).

**Table 1:** Baseline laboratory results. Most parameters were within normal limits; serum ACE was elevated, and IGRA was negative. ACE: angiotensin-converting enzyme; ALP: alkaline phosphatase; ALT: alanine aminotransferase; ANCA: antineutrophil cytoplasmic antibodies; AST: aspartate aminotransferase; CCP: cyclic citrullinated peptide; CK: creatine kinase; ESR: erythrocyte sedimentation rate; GGT: gamma-glutamyltransferase; IGRA: interferon-gamma release assay; MPO: myeloperoxidase; PR3: proteinase 3; RF: rheumatoid factor

Parameter	Measured value	Reference range
Hematology		
Hemoglobin (g/dL)	14.4	12.0-15.0
Leukocytes (×10⁹/L)	5.64	4.5-11.0
Platelets (×10⁹/L)	217	150-450
Renal and liver function		
Creatinine (mg/dL)	0.88	0.51-0.95
Urea (mg/dL)	32	16.6-48.5
Total protein (g/L)	70.8	64-83
Total bilirubin (mg/dL)	0.5	<0.90
AST (U/L)	13	<32
ALT (U/L)	8	<33
GGT (U/L)	18	<40
ALP (U/L)	50	35-104
Inflammatory and muscle markers		
CRP (mg/L)	<0.6	<5.0
ESR (mm/h)	12	<16
CK (U/L)	42	<170
Sarcoidosis-related and infection screening		
ACE (U/L)	70	8-52
IGRA (QuantiFERON)	Negative	Negative
Autoimmune panel		
Rheumatoid factor (IU/mL)	<9.3	<15
Anti-CCP (UQ)	<4.6	<20
ANCA PR3 (UQ)	<2.3	<20
ANCA MPO (UQ)	<3.2	<20

Pulmonary function tests were within normal limits (Table 2). Cardiac MRI excluded myocardial involvement.

**Table 2:** Pulmonary function tests at baseline demonstrating normal spirometry, lung volumes, and lung diffusing capacity. DLCO: diffusing capacity of the lungs for carbon monoxide; FEV₁: forced expiratory volume in one second; FVC: forced vital capacity; KCO: transfer coefficient; RV: residual volume; TLC: total lung capacity

Parameter	Measured value	% Predicted
FEV₁ (L)	4.08	113%
FVC (L)	5.25	117%
TLC (L)	6.9	116%
RV (L)	1.81	110%
DLCO (mmoL/(min*kPa))	7.78	94%
KCO (mmoL/(min*kPa*L))	1.17	84%

The final diagnosis of multisystem sarcoidosis with pulmonary, lymphatic, and axial skeletal involvement was established at a multidisciplinary team meeting.

Treatment was initiated with oral prednisolone 40 mg per day with gradual tapering. Methotrexate was subsequently introduced and titrated to 15 mg per week as a steroid-sparing agent. The patient experienced a marked clinical response within two months, with substantial improvement in fatigue, joint and bone pain, and exercise tolerance. She has since remained clinically stable on methotrexate and low-dose corticosteroids (10 mg per day), with sustained symptomatic benefit for over one year.

**Figure 5:**
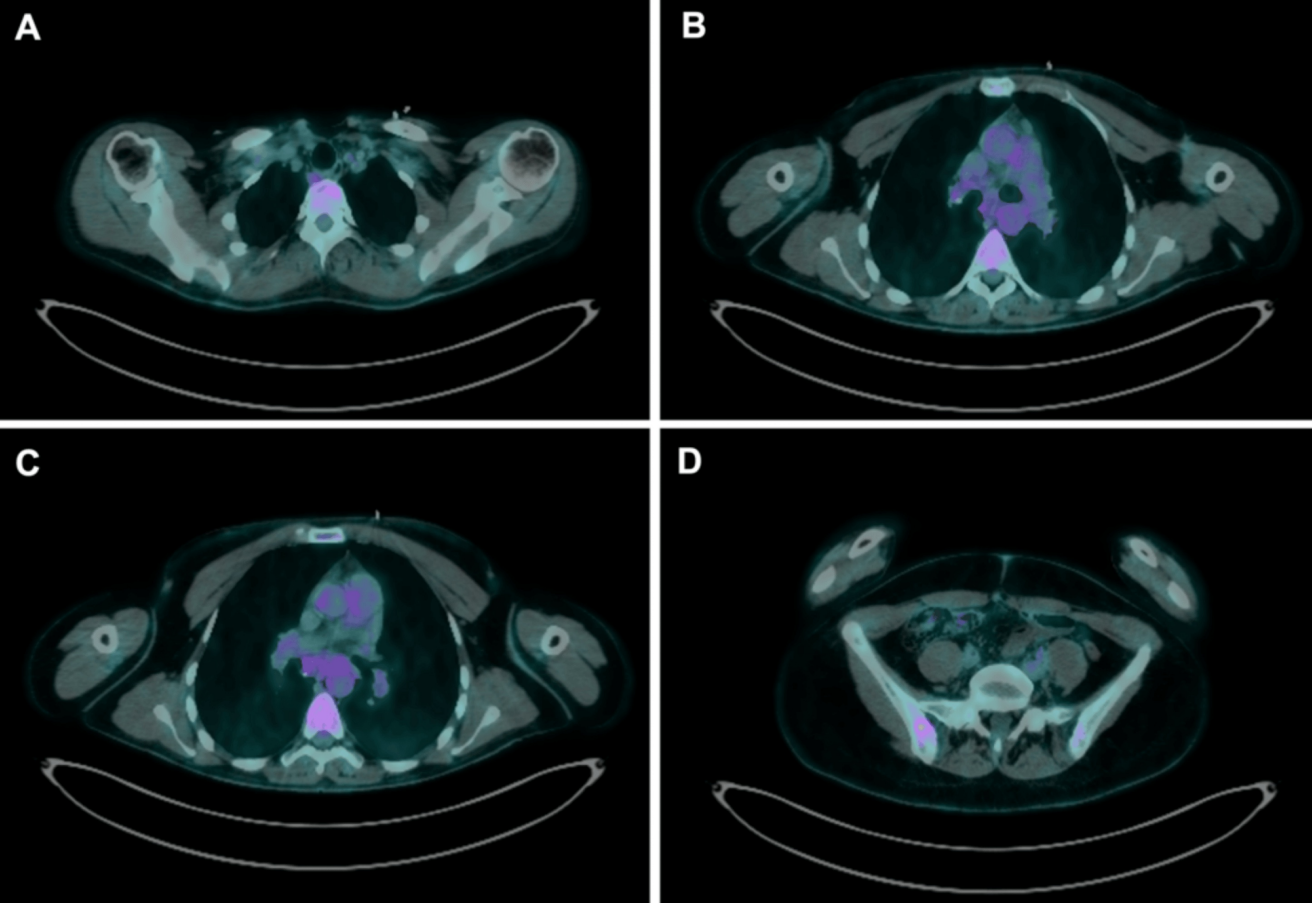
Follow-up ¹⁸F-FDG PET/CT images showing (A) resolution of pulmonary uptake, (B, C) mild residual metabolic activity in mediastinal and hilar lymph nodes (SUVmax = 3.1), and (D) moderate uptake in the right iliac bone (SUVmax = 4.3). ¹⁸F-FDG PET/CT: fluorine-18 fluorodeoxyglucose positron emission tomography/computed tomography

Follow-up ¹⁸F-FDG PET/CT performed one year after treatment initiation demonstrated a marked reduction in metabolic activity of pulmonary, nodal, and osseous lesions, with only mild residual uptake in mediastinal and hilar lymph nodes (SUVmax = 3.1) and a moderate focus in the right iliac bone (SUVmax = 4.3) (Figure 5A-5D), findings consistent with treatment response.

## Discussion

This case describes a challenging presentation of multisystem sarcoidosis with metabolically active FDG-avid pulmonary nodules, nodal involvement, and axial skeletal lesions. Such findings frequently raise suspicion of malignancy and prompt oncologic evaluation. Nevertheless, FDG uptake reflects inflammatory cell glycolysis and is not disease-specific. Granulomatous inflammation may closely mimic malignancy on PET/CT, particularly in bone, thereby reducing specificity and prompting histological confirmation to establish the diagnosis [12-14]. In this case, the initial SUV values in the lung, lymph nodes, ilium, and vertebra were within the ranges reported for both sarcoidosis and metastatic disease, underscoring the need for histopathological confirmation rather than reliance on metabolic intensity alone.

Osseous sarcoidosis is relatively uncommon but may be underrecognized. Earlier prevalence estimates of 3-13% were based on conventional imaging series. With the use of modern imaging modalities such as PET/CT and MRI, axial involvement, particularly of the spine and pelvis, has been detected more frequently than previously appreciated. [4,6,7,15]. In a large cohort, the spine and pelvis were the most affected sites (approximately 69% and 36%, respectively) [4]. More recent PET-based studies suggest that about one-third of patients show axial hypermetabolism, often occult on low-dose CT [6,7,15]. The iliac and vertebral lesions observed in our patient were consistent with these patterns.

Imaging is essential for mapping the extent of disease and selecting biopsy targets. FDG-PET/CT can guide sampling of the most metabolically active site and is useful for assessing inflammatory activity. However, inflammatory and neoplastic processes overlap metabolically, and false positives may occur in infection and inflammation [12,13]. In our case, combined nodal and bone biopsies showing noncaseating granulomatous inflammation established the diagnosis and excluded malignancy and infection, which is aligned with current diagnostic principles that emphasize the need for concordant clinical and radiological findings, histological confirmation, and exclusion of alternative causes [2].

Diagnostic integration within an MDD is considered the gold standard for interstitial lung diseases, improving diagnostic confidence and impacting management [9,16]. Incorporating our patient’s clinical, radiologic, and pathologic data into MDD appropriately supported the diagnosis of multisystem sarcoidosis with osseous involvement and guided therapy selection.

Not all patients require treatment. Decisions depend on organ-threatening disease, functional impairment, or significant symptom burden [10,11]. International guidelines recommend systemic corticosteroids as the initial therapy in many cases. Methotrexate is a common steroid-sparing agent when prolonged treatment is anticipated or when steroid toxicity emerges [1,17]. Evidence, largely from observational cohorts and expert consensus, supports methotrexate as an effective steroid-sparing agent with clinical benefit across organ systems, while highlighting the need for careful monitoring and individualized dosing [1,17,18]. Our patient’s sustained symptomatic improvement and subsequent metabolic response on follow-up PET are consistent with reports that axial bone lesions may show favorable evolution under anti-inflammatory therapy, with PET/MRI often demonstrating interval resolution or marked reduction in activity [15].

Finally, laboratory markers such as serum ACE may support disease activity assessment, but their diagnostic accuracy is limited by variable sensitivity and specificity and by elevations in conditions other than sarcoidosis. This reinforces their adjunctive rather than definitive role, emphasizing the importance of tissue confirmation and multidisciplinary decision-making in cases with ambiguous presentation mimicking malignancy [2,19,20].

## Conclusions

Sarcoidosis is a multisystem granulomatous disease with diverse clinical manifestations. Axial skeletal involvement is uncommon and can closely resemble metastatic disease, often prompting extensive oncologic investigation. This case underscores the importance of considering sarcoidosis in the differential diagnosis of hypermetabolic bone lesions and highlights the critical role of histological confirmation and MDD in establishing an accurate diagnosis. The favorable clinical and metabolic response to corticosteroids and methotrexate further illustrates the value of individualized immunosuppressive therapy and reinforces the need for a tailored, evidence-based approach in managing complex presentations of this disease.
